# A novel simple disposition index (SPINA‐DI) from fasting insulin and glucose concentration as a robust measure of carbohydrate homeostasis

**DOI:** 10.1111/1753-0407.13525

**Published:** 2024-01-02

**Authors:** Johannes W. Dietrich, Assjana Abood, Riddhi Dasgupta, Shajith Anoop, Felix K. Jebasingh, R. Spurgeon, Nihal Thomas, Bernhard O. Boehm

**Affiliations:** ^1^ Diabetes, Endocrinology and Metabolism Section, Department of Internal Medicine I, St. Josef Hospital Ruhr University Bochum Bochum Germany; ^2^ Diabetes Centre Bochum/Hattingen, St. Elisabeth‐Hospital Blankenstein Hattingen Germany; ^3^ Centre for Rare Endocrine Diseases, Ruhr Centre for Rare Diseases (CeSER) Ruhr University Bochum and Witten/Herdecke University Bochum Germany; ^4^ Centre for Diabetes Technology Catholic Hospitals Bochum Bochum Germany; ^5^ Department of Endocrinology, Diabetes and Metabolism Christian Medical College Vellore India; ^6^ Department of Endocrinology Bangalore Baptist Hospital Bangalore India; ^7^ Lee Kong Chian School of Medicine Nanyang Technological University Singapore Singapore Singapore; ^8^ King's College London School of Life Course & Population Sciences London UK

**Keywords:** disposition index, dynamical compensation, insulin‐glucose homeostasis

## Abstract

**Aims:**

The widely used dynamic disposition index, derived from oral glucose tolerance testing, is an integrative measure of the homeostatic performance of the insulin‐glucose feedback control. Its collection is, however, time consuming and expensive. We, therefore, pursued the question if such a measure can be calculated at baseline/fasting conditions using plasma concentrations of insulin and glucose.

**Methods:**

A new fasting‐based disposition index (structure parameter inference approach‐disposition index [SPINA‐DI]) was calculated as the product of the reconstructed insulin receptor gain (SPINA‐GR) times the secretory capacity of pancreatic beta cells (SPINA‐GBeta). The novel index was evaluated in computer simulations and in three independent, multiethnic cohorts. The objectives were distribution in various populations, diagnostic performance, reliability and correlation to established physiological biomarkers of carbohydrate metabolism.

**Results:**

Mathematical and in‐silico analysis demonstrated SPINA‐DI to mirror the hyperbolic relationship between insulin sensitivity and beta‐cell function and to represent an optimum of the homeostatic control. It significantly correlates to the oral glucose tolerance test based disposition index and other important physiological parameters. Furthermore, it revealed higher discriminatory power for the diagnosis of (pre)diabetes and superior retest reliability than other static and dynamic function tests of glucose homeostasis.

**Conclusions:**

SPINA‐DI is a novel simple reliable and inexpensive marker of insulin‐glucose homeostasis suitable for screening purposes and a wider clinical application.

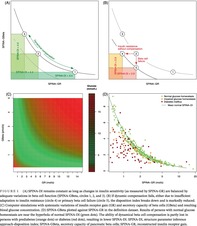

## Graphical abstract

Highlights

- We defined a fasting‐based disposition index (structure parameter inference approach‐disposition index [SPINA‐DI]), calculated as the product of the secretory capacity of pancreatic beta cells (SPINA‐GBeta) multiplied by estimated insulin receptor gain (SPINA‐GR).
- SPINA‐DI mirrors the recognized hyperbolic relationship between beta‐cell function and insulin sensitivity.
- It significantly correlates with the dynamic disposition index based on frequently sampled oral glucose tolerance tests (Matsuda–DeFronzo method) and other important measures of insulin‐glucose homeostasis.
- For the diagnosis of diabetes and prediabetes, it has a higher discriminatory power than other static function tests (homeostatic model assessment of β‐cell function, homeostatic model assessment of insulin resistance, and quantitative insulin sensitivity check index) and dynamic, oral glucose tolerance testing‐derived parameters (insulin sensitivity index, insulinogenic index and disposition index according to Matsuda and DeFronzo).
- SPINA‐DI has high retest reliability compared to other markers of glucose homeostasis.
- This novel fasting‐based disposition index (SPINA‐DI) is defined as the product of insulin receptor gain (SPINA‐GR) multiplied by beta‐cell function (SPINA‐GBeta). SPINA‐DI may be reduced due to insulin resistance with insufficient dynamical compensation of beta‐cell mass (red horizontal arrow) or due to primary beta‐cell failure (red vertical arrow). SPINA‐DI was validated in three cohorts. It had advantages in validity, reliability, and diagnostic power compared to established criteria.

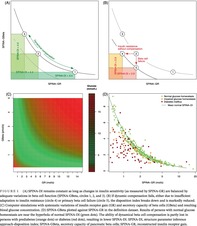

## 1 INTRODUCTION

In healthy conditions, the pancreatic islet cells are able to adapt to variations in insulin sensitivity. This behavior, a classic example of dynamical compensation,^1^ helps to mitigate the effects of varying insulin sensitivity in short‐ and long‐term time scales.^2^ Therefore, insulin‐resistant subjects are able to maintain normal blood glucose concentrations as long as they are able to sustain high plasticity in response to metabolic challenges by increasing the mass and secretory capacity of beta cells.^2^

For diagnostic purposes, dynamical compensation renders the interpretation of beta‐cell function purposes challenging, because a similar degree of beta‐cell output may be considered as normal or inappropriate, depending on the level of insulin sensitivity.^3^, ^4^, ^5^ Likewise, insulin sensitivity alone cannot be readily used to assess the function of glucose homeostasis without simultaneously considering the extent of beta‐cell function.^6^ Therefore, more comprehensive disposition metrics are required and may be performed by integrating the two main processes of the feedback circuit.^7^, ^8^

In control systems technology and electronics, calculating the loop gain, that is, the product of the gains of all elements that form a feedback loop has been proven useful.^9^ The loop gain (as an equivalent to the functional compensation of beta cells) describes the amplification of a signal that passes once through the feedback circuit. It determines the performance and stability of the control system.

For the assessment of insulin‐glucose homeostasis, the disposition index (DI) serves a similar purpose. Analogously to the loop gain, it is defined as the product of beta‐cell function times insulin sensitivity.^10^ Reductions in the DI, which is driven by endoplasmic reticulum stress, mitochondrial dysfunction, oxidative stress, and inflammation at the level of the beta cell, predict the risk of developing (pre)diabetes.^2^, ^11^

Usually, the DI is calculated from dynamical function tests, including frequently sampled oral glucose tolerance testing (OGTT) and glucose clamp procedures.^12^ Although efforts have been undertaken to reduce the number of time points to calculate the composite index^13^ these procedures remain elaborate and expensive. Especially for screening purposes as well as for clinical practice, approaches based on fasting concentrations of insulin and glucose without requiring dynamical function tests may be advantageous. Moreover, fasting‐based markers could provide valuable additional information on the biology of the feedback loop, because the static and dynamic behavior of pancreatic beta cells are governed by different properties.^14^ Consequently, we have conceived a novel method to calculate a DI from a single blood sample that has been obtained under fasting conditions.

Our work assumes that—despite some differences—both the static and dynamic behavior of the feedback loop have key physiological foundations in common. For instance, pancreatic beta‐cell function will depend on beta‐cell mass,^15^ and this link will apply to both fasting conditions and the response to a glucose load. Likewise, insulin sensitivity is determined by several processes, which include insulin receptor density, mechanisms of signal transduction, and properties of the metabolic machinery, and this again will affect both the fasting and the postabsorptive state. Accordingly, we hypothesized that it would be possible to derive a DI from the fasting steady‐state concentrations that can also predict the behavior after a glucose load.

The objectives for a useful fasting‐based disposition metric include the following criteria:

- It should be based on validated measures of insulin sensitivity and beta‐cell function.
- It should be invariant with respect to a given range of insulin sensitivity, that is, over the range of population means, a certain degree of insulin sensitivity should deliver a reproducible degree of disposition if combined with the corresponding quantity of beta‐cell function.
- It should represent the known hyperbolic relationship between beta‐cell function and insulin sensitivity.
- It should reliably distinguish between subjects with and without diabetes.
- It should correlate with established biomarkers of insulin‐glucose homeostasis.

## 2 METHODS

### 2.1 Development of the technique

The novel fasting‐based DI is based on the structure parameter inference approach (SPINA‐Carb), a validated model‐based static function test of insulin‐glucose homeostasis.^16^ This method builds upon a nonlinear mathematical theory of the feedback control system and delivers two calculated markers of the homeostatic system, the secretory capacity of beta‐cells (SPINA‐GBeta) and the insulin receptor gain (SPINA‐GR). In accordance with other definitions of disposition metrics, the new fasting‐based DI is calculated as the product of insulin sensitivity times beta‐cell output with

$$\text{SPINA}–\mathrm{DI}=\text{SPINA}–G_{R}∙\text{SPINA}–G_{\beta }$$

Detailed information on the calculation of SPINA‐GR and SPINA‐GBeta is provided in the Data S1.

In a first in silico analysis, the new index was validated in computer simulations. For this purpose, two‐way sensitivity analysis was carried out with SimulaBeta 3.1^17^ by systematically varying the parameters GR and GBeta and recording the consequences for steady‐state glucose concentration.

### 2.2 Cohorts for definition and validation

The new index was evaluated in three independent cohorts from three continents, spanning various ethnic groups, metabolic challenges, nutritional status, and comorbidities. The three datasets were analyzed separately and served as definition, validation, and test datasets, similar to the recommended practice for machine learning and data mining.

The first dataset was derived from the 2009/2010 period of the US National Health and Nutrition Examination Survey (NHANES), a large research program to assess the health and nutritional status of the population in the United States. It served as definition dataset to obtain population‐based distributions and a preliminary reference range. It was also used for the analysis of potential correlations of anthropometric data and OGTT with SPINA‐DI. A second cohort, serving as validation dataset, included subjects from a previous case–control sequencing study for type 2 diabetes.^18^ All participants were residents of Germany with and without diabetes, who underwent frequently sampled (OGTT) with determinations of glucose, insulin, c‐peptide, proinsulin, and adipocytokine concentrations. A third dataset (test dataset) was obtained from the original definition study for SPINA‐GR and SPINA‐GBeta.^16^ It included an independent cohort from rural Southern India, based on observations of anthropometric measurements and OGTT in a homogeneous cohort of normoglycemic Asian Indian males with low body mass index (BMI). The validation and test datasets were used to verify the agreement between SPINA‐DI and the OGTT‐based DI according to Matsuda and DeFronzo and to test correlations with several biomarkers of insulin‐glucose homeostasis.

The inclusion criteria for the definition dataset were the availability of data on OGTT and the presence of anthropometric measurements (body mass and length) as well as fasting glucose and insulin concentrations. Exclusion criteria were treatment with insulin or oral antidiabetic agents. For obtaining reference ranges for SPINA‐GR, SPINA‐GBeta, and SPINA‐DI, a subgroup of nonpregnant persons with no history of diabetes, prediabetes, or thyroid disease; sex‐specific normal BMI; fasting glucose concentration < 5.6 mmol/L; 2‐h glucose concentration in OGTT <7.8 mmol/L; and glycated hemoglobin (HbA1c) < 5.7% was selected.

The inclusion criteria for the validation dataset were a family background of metabolic disorders including obesity, metabolic syndrome, and diabetes. The exclusion criteria were the presence of long‐standing diabetes and treatment with insulin, incretin mimetics, oral antidiabetic agents, and glucocorticoids.

The test dataset included OGTT studies from nonobese (BMI < 20 kg/m^2^) and young Asian Indian persons from Southern India. This study included males aged between 18 and 22 years who were healthy, normoglycemic, normolipidemic individuals with no history of smoking or alcoholism, hepatic diseases, HIV, malignancies of any form, and drug overuse. They were not on any medications or supplements that could interfere with the results of insulin or glucose determinations. In order to obtain information on retest reliability and intraindividual clustering, repeated measurements of fasting insulin and glucose concentrations were performed with an interval of 4 days between repeats.

### 2.3 Statistical methods

All statistical analyses were performed with custom S scripts written for the environment Using R 4.2.3 on macOS.^19^ Reference ranges for SPINA‐GR, SPINA‐GBeta, and SPINA‐DI were obtained from 95% tolerance intervals based on continuous sample quantiles type 8 as recommended by Hyndman and Fan.^20^ Biomarkers in different groups (no diabetes, prediabetes, and diabetes) were compared via Kruskal–Wallis tests and post‐hoc pairwise Wilcoxon‐Mann–Whitney *U*‐test. Alpha error correction for multiple testing was performed with the Benjamini–Hochberg procedure. Correlations between continuous variables were evaluated with Spearman's rank correlation. Ergodicity of biomarkers was evaluated as repeatability from intraindividual and interindividual variances with

$$e=\frac{{\mathrm{Var}}_{\text{interindividual}}}{{\mathrm{Var}}_{\text{intraindividual}}+{\mathrm{Var}}_{\text{interindividual}}}$$

and with Spearman's rho. Assessment of diagnostic utility was performed with receiver operating characteristic analysis to yield sensitivity and specificity ranges for each parameter. In places not otherwise specified, the data are presented as mean value and SEM. A *p* < .05 was considered statistically significant.

### 2.4 Ethics approval and consent to participate

The NHANES protocol (definition cohort) has been approved by the National Center for Health Statistics Research Ethics Review Board of the US National Center for Health Statistics (continuation of protocol #2005–06). The study protocol of the validation dataset was approved by the Institutional Review Board of the University of Ulm with the registration number (Ulm DM studies: 42/2004, 189/2007). The protocol for the OGTT study for the test dataset was approved by the Institutional Review Board of Christian Medical College, Vellore, India (Research Committee Minute Number: 5879, 2006 and Administrative Committee Minute Number: 50‐y: 6–2006). Informed consent was obtained from all subjects, prior to inclusion in the study.

All research has been performed in accordance with the Declaration of Helsinki.

## 3 RESULTS

### 3.1 Mathematical properties of SPINA‐DI

**FIGURE 1:**
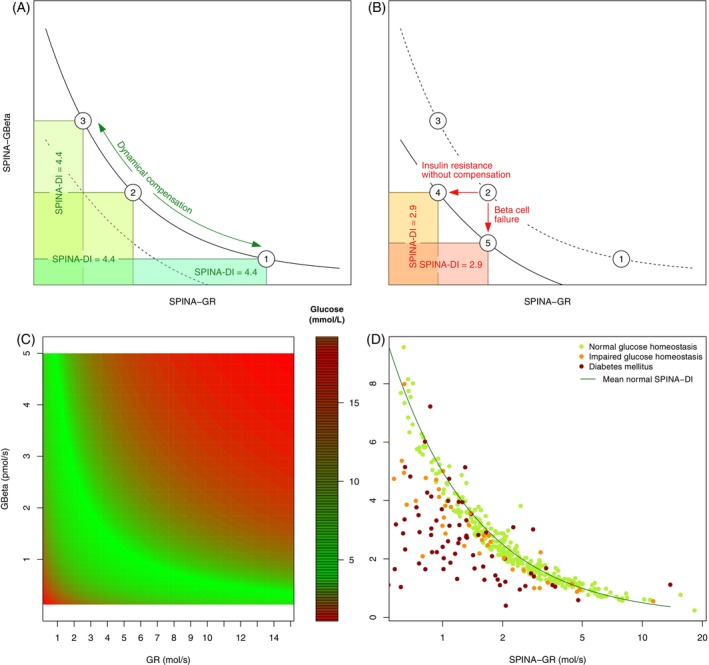
(A) SPINA‐DI remains constant as long as changes in insulin sensitivity (as measured by SPINA‐GR) are balanced by adequate variations in beta cell function (SPINA‐GBeta, circles 1, 2, and 3). (B) If dynamic compensation fails, either due to insufficient adaptation to insulin resistance (circle 4) or primary beta cell failure (circle 5), the disposition index breaks down and is markedly reduced. (C) Computer simulations with systematic variations of insulin receptor gain (GR) and secretory capacity of beta cells (GBeta) and resulting blood glucose concentration. (D) SPINA‐GBeta plotted against SPINA‐GR in the definition dataset. Results of persons with normal glucose homeostasis are near the hyperbola of normal SPINA‐DI (green dots). The ability of dynamical beta cell compensation is partly lost in persons with prediabetes (orange dots) or diabetes (red dots), resulting in lower SPINA‐DI. SPINA‐DI, structure parameter inference approach‐disposition index; SPINA‐GBeta, secretory capacity of pancreatic beta cells; SPINA‐GR, reconstructed insulin receptor gain.

By definition as a product of SPINA‐GR and SPINA‐GBeta, the fasting‐based DI reproduces the hyperbolic relationship between insulin sensitivity and beta‐cell function (Figure 1). Therefore, the product remains invariant as long as dynamical compensation, composed of increased beta‐cell mass, unfolded protein response, and improving mitochondrial function, adjusts SPINA‐GBeta to variations in SPINA‐GR (Figure 1A). Mechanisms leading to diabetes have a marked influence on SPINA‐DI that is clearer than that on SPINA‐GBeta or SPINA‐GR (Figure 1B).

Two‐way sensitivity analysis derived from computer simulations confirms that combinations of GBeta and GR that follow a hyperbolic relationship deliver optimal glucose concentrations (Figure 1C).

### 3.2 Subject characteristics

**FIGURE 2:**
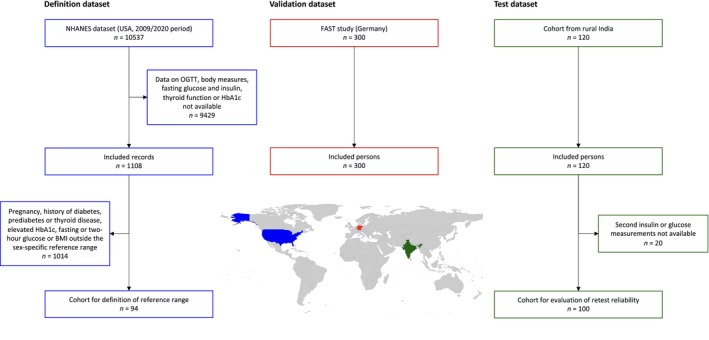
Flow chart of the three cohorts. The numbers of eligible, excluded, enrolled, and analyzed persons are shown. BMI, body mass index; FAST, study on *fast* insulin response in OGTT; HbA1c, glycated hemoglobin; NHANES, National Health and Nutrition Examination Survey; OGTT, oral glucose tolerance test.

A total of 1528 persons were included in the three cohorts. Among them, 336 were affected by newly diagnosed diabetes mellitus, 45 by prediabetes, and 1130 were nondiabetic subjects. The study flow chart is represented in Figure 2. Basic clinical features of the study populations are reported in Table 1.

**TABLE 1:** Clinical characteristics of the three studied cohorts.

	Definition dataset (NHANES 2009/2010 cohort, *n* = 1108)	Validation dataset (OGTT study in German persons, *n* = 300)	Test dataset (OGTT study in Asian Indian males, *n* = 120)
Age (years)	42.8 ± 7.1	49.9 ± 11.4	19.8 ± 1.0
Sex			
Female	549	124	0
Male	599	167	120
Body surface area, m^2^	1.9 ± 0.3	N/A	1.7 ± 0.1
Body mass index, kg/m^2^	28.2 ± 7.1	26.8 ± 1.2	19.3 ± 2.8
Waist circumference, cm	95.8 ± 17.0	N/A	70.1 ± 7.4
Fat‐free mass, kg	N/A	N/A	43.4 ± 5.4
Triceps skinfold, mm	18.6 ± 8.0	N/A	N/A
Subscapular skinfold, mm	19.7 ± 8.4	N/A	N/A
Diabetes mellitus			
Normal glucose homeostasis	947	63	120
Prediabetes	45	0	0
Diabetes	99	237^a^	0
Fasting glucose (mmol/L)	5.7 ± 1.5	7.7 ± 2.6	5.0 ± 0.5
Fasting insulin (mmol/L)	68.6 [59.6]	66.0 [84.0]	19.2 [15.2]
HbA1c (%)	5.6 ± 0.9	N/A	5.3 ± 0.4

*Note*: Data are reported as mean ± SD, median [interquartile range] or counts (percentage).

Abbreviations: HbA1c, glycated hemoglobin; OGTT, oral glucose tolerance test; N/A, data not available; NHANES, US National Health and Nutrition Examination Survey.

^a^ Newly diagnosed diabetes.

For nondiabetic subjects, preliminary reference ranges for SPINA‐GBeta, SPINA‐GR, and SPINA‐DI could be obtained (first column of Table 2).

**TABLE 2:** Measured and calculated parameters of the feedback loop in subjects with diabetes mellitus, prediabetes and normal glucose homeostasis.

Parameter (Reference range)	Definition dataset			Validation dataset	
	Normal (*n* = 947)	Prediabetes (*n* = 45)	Diabetes (*n* = 99)	Normal (*n* = 63)	Diabetes (*n* = 237)
Fasting glucose (<5.6 mmol/L)	5.58 ± 0.04	6.42 ± 0.38***	7.82 ± 0.31^†††###^	5.41 ± 0.22	8.35 ± 0.16^†††^
Fasting insulin (12–150 pmol/L)	80.6 ± 2.2	91.9 ± 8.5	112.5 ± 20.7*	54.8 ± 5.6	100.5 ± 5.2^††^
Two‐hour glucose in OGTT (<7.8 mmol/L)	6.47 ± 0.12	8.31 ± 0.80	8.59 ± 3.63**	6.81 ± 0.27	14.84 ± 0.41^†††^
ISI	N/A	N/A	N/A	8.22 ± 0.75	3.66 ± 0.25^††^
IGI (pmol/mol)	N/A	N/A	N/A	0.28 ± 0.05	0.14 ± 0.02***
Disposition index (Matsuda and DeFronzo)	N/A	N/A	N/A	1.58 ± 0.23	0.37 ± 0.07^††^
HOMA‐Beta	140.9 ± 7.8	129.7 ± 12.0	118.6 ± 15.6^†#^	116.1 ± 10.9	95.8 ± 62.9**
HOMA‐IR (<2.5)	3.5 ± 0.1	4.4 ± 0.5*	7.1 ± 1.7^††^	2.3 ± 0.3	6.4 ± 0.4^†††^
HOMA‐IS	0.49 ± 0.02	0.41 ± 0.07*	0.31 ± 0.03^††^	0.75 ± 0.06	0.34 ± 0.03^††^
QUICKI (>0.4)	0.33 ± 0.00	0.32 ± 0.01*	0.31 ± 0.00^††^	0.36 ± 0.00	0.31 ± 0.00^†††^
SPINA‐GBeta (0.64–3.73 pmol/s)^§^	3.07 ± 0.08	3.50 ± 0.29	3.66 ± 0.58	2.15 ± 0.21	3.21 ± 0.16***
SPINA‐GR (1.41–9.00 mol/s)^§^	2.15 ± 0.07	1.78 ± 0.30*	1.27 ± 0.16^†††#^	3.33 ± 0.27	1.33 ± 0.11^†††^
SPINA‐DI (4.01–7.65)^§^	4.29 ± 0.04	3.78 ± 0.18**	2.91 ± 0.21^†††‡‡^	4.77 ± 0.16	2.44 ± 0.10^†††^

*Note*: *p < .05, **< .01, ***< .001, ^†^ < 1e–4, ^††^ < 1e–5, ^†††^ 1e–10 compared to subjects with normal glucose homeostasis. ^#^ *p* < .05, ^##^ < .01, ^###^ < .001, ^‡^ < 1e–4, ^‡‡^ < 1e–5, ^‡‡‡^ < 1e–10 compared to subjects with prediabetes. ^§^Reference range derived from definition dataset in this study.

Abbreviations: HOMA‐Beta, homeostatic model assessment of β‐cell function; HOMA‐IR, homeostatic model assessment of insulin resistance; HOMA‐IS, homeostatic model assessment for insulin sensitivity; IGI, insulinogenic index; ISI, insulin sensitivity index; OGTT, oral glucose tolerance test; QUICKI, quantitative insulin sensitivity check index; SPINA‐GBeta, secretory capacity of pancreatic beta cells; SPINA‐GR, reconstructed insulin receptor gain; SPINA‐DI, structure parameter inference approach‐disposition index.

### 3.3 Comparing subjects with and without diabetes

In both the definition and the validation dataset key markers of insulin‐glucose homeostasis differed between subjects with and without diabetes. In the definition dataset distributions of subjects with prediabetes could be obtained as well (Table 2).

For nondiabetic persons, the pairs of SPINA‐GBeta and SPINA‐GR are located in the vicinity of the hyperbola defined by normal SPINA‐DI (Figure 1D).

**FIGURE 3:**
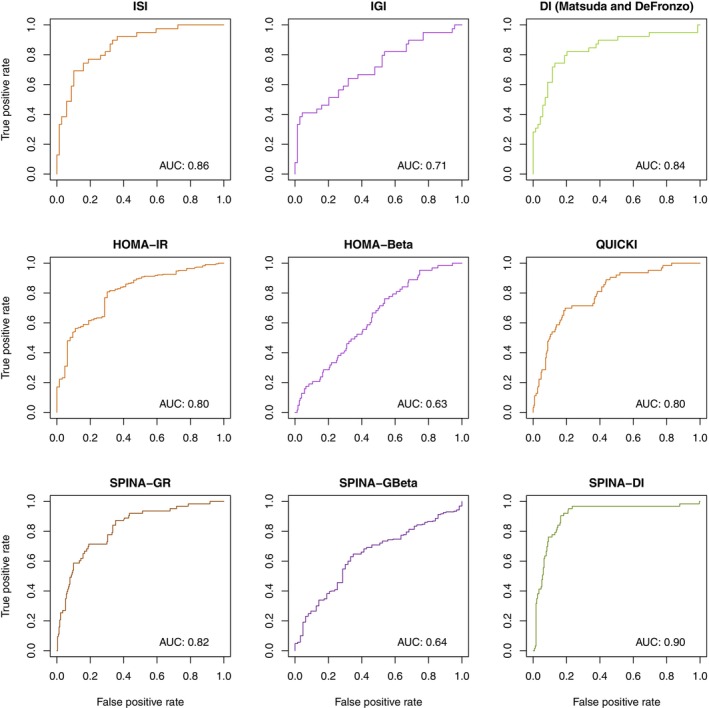
SPINA‐DI has high discriminatory power for the distinction between diabetes and normal glucose homeostasis. Receiver operating characteristic (ROC) curves and corresponding areas under the curve (AUC) are shown for several markers for the function of the insulin‐glucose feedback control in the validation dataset. HOMA‐Beta, homeostatic model assessment of β‐cell function; HOMA‐IR, homeostatic model assessment of insulin resistance; IGI, insulinogenic index; ISI, insulin sensitivity index; QUICKI, quantitative insulin sensitivity check index; SPINA‐DI, structure parameter inference approach‐disposition index; SPINA‐GBeta, secretory capacity of pancreatic beta cells; SPINA‐GR, reconstructed insulin receptor gain.

Compared to healthy persons, SPINA‐GR was lower in subjects with diabetes and prediabetes, SPINA‐GBeta tended to be higher, and SPINA‐DI was significantly reduced (Figure S1). SPINA‐DI had a higher discriminatory power than other calculated and OGTT‐derived parameters (Figure 3).

### 3.4 Correlations to metabolic biomarkers

**FIGURE 4:**
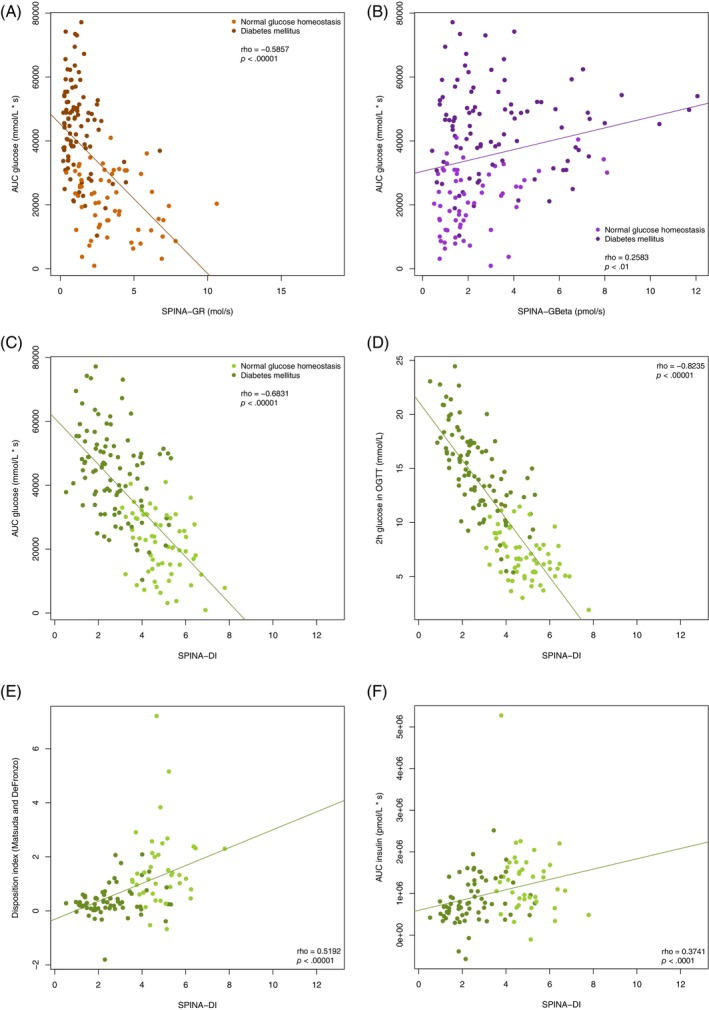
SPINA‐GR (A), SPINA‐GBeta (B), and SPINA‐DI (C) correlate with the area under the glucose curve in oral glucose tolerance testing and with the glucose concentration after 2 h (D). SPINA‐DI also correlates to the disposition index according to Matsuda and DeFronzo (E) and to the area under the insulin curve in oral glucose tolerance test (OGTT) (F). Areas under the curve are baseline corrected. AUC, area under the curve; SPINA‐DI, structure parameter inference approach‐disposition index; SPINA‐GBeta, secretory capacity of pancreatic beta cells; SPINA‐GR, reconstructed insulin receptor gain.

The fasting‐based DI significantly correlates with the dynamical DI according to Matsuda and DeFronzo, the 2‐h glucose concentration and the area under the curve of oral glucose tolerance testing (Figure 4C–F). The correlations are stronger than those of SPINA‐GR and SPINA‐GBeta (Figure 4A,B).

SPINA‐DI also significantly correlates with HbA1c, BMI, waist circumference, and subscapular skinfold in the definition dataset; 2‐h OGTT concentrations of proinsulin and c‐peptide and concentrations of free fatty acids, ghrelin, and adiponectin in the validation dataset; and the insulin sensitivity index (ISI) in the test dataset. Correlations with age in the definition dataset could not be reproduced in the test dataset.

Comprehensive correlation networks are shown in Figure S2. In all three datasets, the 2‐h glucose concentration, the area under the curve in OGTT, and the HbA1c concentration showed a stronger correlation to SPINA‐DI than to other calculated parameters including SPINA‐GBeta, SPINA‐GR, homeostatic model assessment of β‐cell function (HOMA‐Beta), homeostatic model assessment of insulin resistance (HOMA‐IR), and quantitative insulin sensitivity check index.

### 3.5 Retest reliability and ergodicity

SPINA‐DI has high retest reliability, as quantified with *e* from results in repeated measurements (Table S2). Spearman's *rho* is higher than that of all other calculated parameters.

## 4 DISCUSSION

### 4.1 Main findings

We have developed a novel disposition metric for insulin‐glucose homeostasis. It is based on two key parameters of the insulin‐glucose feedback loop, namely insulin sensitivity (SPINA‐GR) and beta‐cell function (SPINA‐GBeta). This novel DI (SPINA‐DI) takes advantage of the fact that it can be obtained from fasting concentrations of insulin and glucose obviating the need for complex dynamical function tests.

SPINA‐DI reproduces the hyperbolic relationship between insulin sensitivity and beta‐cell function. Under circumstances with fully functional dynamical metabolic compensation, it remains constant over the range of insulin resistance and consecutive adaptation of beta‐cell mass. It correlates with pertinent measures of metabolic condition including BMI, waist circumference, and subscapular skinfold as well as concentrations of ghrelin, adiponectin, and free fatty acids. Additionally, it predicts important results of OGTT including the glucose concentration after 2 h, the area under the glucose curve, and the OGTT‐based DI according to Matsuda and DeFronzo. It significantly differs between distinct metabolic entities such as normal glucose homeostasis, prediabetes, and diabetes, and its discriminatory power is higher than that of other calculated and/or OGTT‐based metrics. Additionally, its test–retest reliability is stronger compared to other markers of homeostatic ß cell function.

### 4.2 Clinical implications

The diagnosis of diabetes mellitus is based on fasting glucose concentration, glucose response to OGTT, and HbA1c. High‐risk stages of diabetes development such as metabolic syndrome, prediabetes, and polycystic ovary syndrome are preferably identified during the long compensatory period to facilitate protective interventions in a timely manner.^21^, ^22^, ^23^, ^24^ Even more advantageous are physiological parameters that can be mapped to meaningful pathogenetic processes of insulin sensitivity and beta‐cell function, as demonstrated for the homeostatic model assessment^25^, ^26^ or the OGTT‐based DI according to Matsuda and DeFronzo.^27^, ^28^ Recently, it could be shown that HOMA may be used for the cluster‐based subclassification of prediabetes and type 2 diabetes as well.^29^, ^30^, ^31^

However, the diagnostic utility of the HOMA‐based biomarkers is limited,^16^, ^32^ and obtaining the OGTT‐based DI is time consuming and expensive. These elements preclude in particular precision diagnostic approaches pinpointing the compensatory and the early phase of dysfunctional ß‐cell function in countries with the highest rise in (pre)diabetes.^33^, ^34^

A recently developed nonlinear approach (SPINA Carb) circumvents some of the disadvantages of the HOMA method, but it does not provide an integrative measure for the quality of the feedback loop.^16^ Therefore, we developed a new composite marker (SPINA‐DI) defined by the product of SPINA‐GBeta times SPINA‐GR. It shares some conceptual analogies to both the loop gain that is used in control systems technology and the OGTT‐based DI. Compared to other static and dynamical function tests SPINA‐DI correlates better to meaningful responses in OGTT testing, has a higher discriminatory power for the diagnosis of diabetes, and has higher retest reliability.

In the NHANES study, the diabetes type is not specified. Because subjects on insulin were excluded from the analysis, we may reasonably assume that the included persons with diabetes are in the large majority affected by type 2 diabetes. In these cases, insulin sensitivity, as quantified via SPINA‐GR, was reduced, whereas beta‐cell function was quite high within the reference range of SPINA‐GBeta. This suggests partial and insufficient dynamical compensation.^35^ The lack of compensation is underpinned by the reduced fasting‐based DI.

### 4.3 Advantages and limitations

The novel DI has the benefit of resulting from a simple calculation based on physiologically derived structure parameters of the feedback loop. In the current project, we defined and validated the parameter in three independent cohorts spanning a wide range of genetic, cultural, and phenotypic variation. We were able to assess the physiological validity, reliability, and diagnostical utility of SPINA‐DI.

The limitations of our study include the unavailability of important physiological parameters in the validation dataset and the low sample size of the test dataset resulting into a lower statistical power (see Figure S2C).

The correlation of SPINA‐DI with the OGTT‐based disposition index according to Matsuda and DeFronzo is significant and in positive direction. However, the information delivered by SPINA‐DI is different from the information provided by the Matsuda index, as demonstrated by a quite high dispersion. This implies some differences between fasting and postabsorptive conditions, despite striking similarities. SPINA‐DI should be therefore regarded as a complement to rather than a replacement of the Matsuda index. It may provide valuable information for endotyping the state of glucose homeostasis and may turn out to be more representative of dynamic and static responses of pancreatic beta cells being modulated by different processes.^14^

Similar considerations apply to SPINA‐GBeta and SPINA‐GR that significantly correlate with the insulinogenic index and ISI but show considerable dispersion as well. Carefully planned physiological studies including compartment analysis and determination of intermediate metabolic products are necessary to obtain more clarity about the similarities and discordances between the results of static and dynamical function tests.

### 4.4 Conclusion and perspective

SPINA‐DI is a novel simple integrative parameter of glucose homeostasis that is obtained by simple measurements of fasting insulin and glucose concentrations. In our cohorts, it has higher reliability and diagnostic utility than previously established methods. If the results are confirmed in future studies SPINA‐DI may become a valuable cost‐effective tool for clinical research as well as future predictive, preventive, and personalized approaches in patient care.

## AUTHOR CONTRIBUTIONS

Johannes W. Dietrich and Assjana Abood conceptualized the mathematical methodology. Johannes W. Dietrich and Bernhard O. Boehm conceived and designed the study. Bernhard O. Boehm planned the study of the validation cohort. Riddhi Dasgupta, Shajith Anoop, Felix K. Jebasingh, R. Spurgeon, and Nihal Thomas planned and conducted the clinical investigation in the test cohort. Johannes W. Dietrich and Assjana Abood analyzed and interpreted the data. Johannes W. Dietrich developed software for analysis. Johannes W. Dietrich, Assjana Abood, and Bernhard O. Boehm drafted a first version of the manuscript. All authors read and revised the manuscript and approved the final version.

## FUNDING INFORMATION

Bernhard O. Boehm was supported by the Ministry of Education Singapore, DFG GRK 1041, and Visiting Professorship at Ulm University, Ulm, Germany. Publication fees were partly defrayed by the Open Access Publication Funds of the Ruhr‐Universität Bochum. Open Access funding enabled and organized by Projekt DEAL.

## DISCLOSURE

Johannes W. Dietrich received funding and personal fees from Novo Nordisk, VitalAire, Abbott, Medtronic, Oviva, Egetis Therapeutics, myhomecare, aidhere, Ascensia Diabetes Care, Sanofi‐Henning, Hexal AG, Bristol‐Myers Squibb, and Pfizer and is co‐owner of the intellectual property rights for the patent “System and Method for Deriving Parameters for Homeostatic Feedback Control of an Individual” (Singapore Institute for Clinical Sciences, Biomedical Sciences Institutes, Application Number 201208940‐5, WIPO number WO/2014/088516). All other authors declare no competing interests.

## Supporting information


**Data S1.** Supporting Information.
