# Supplementary material for: A novel simple disposition index (SPINA‐DI) from fasting insulin and glucose concentration as a robust measure of carbohydrate homeostasis
Source: J Diabetes. 2024 Jan 2;16(9):e13525. doi: 10.1111/1753-0407.13525 (PMC11418405; doi:10.1111/1753-0407.13525)
Supplement: Supplementary file 1 — Data S1. Supporting Information. [file JDB-16-e13525-s001.pdf]

Journal of Diabetes Open Access

Supplementary Appendix accompanying the manuscript „ A novel simple disposition index (SPINA-DI) from fasting insulin and glucose concentration as a robust measure of carbohydrate homeostasis"

Johannes W. Dietrich, Assjana Abood, Riddhi Dasgupta, Shajith Anoop, Felix Jebasingh, R. Spurgeon, Nihal Thomas, Bernhard O. Boehm

Supplementary Methods

Calculations

SPINA-GBeta and SPINA-GR are calculated with

$$SPINA\text{-}GBeta = \frac{[I](D_\beta+[G])}{G_3[G]} \quad (1)$$

and

$$SPINA\text{-}GR = \frac{G_1 P(D_R+[I])}{G_E[I][G]} - \frac{D_R}{G_E[I]} - \frac{1}{G_E} \quad (2)$$

from fasting concentrations of insulin ([*I*]) and glucose ([*G*]) and several constants (Table S1) as previously described[1].

From this, SPINA-DI is calculated with

$$SPINA\text{-}DI = SPINA\text{-}G_R \cdot SPINA\text{-}G_\beta. \quad (3)$$

Table S1: Parameters for calculation[1]

Parameter	Explanation	Value
α_G	Dilution factor ($1/V_D$) for glucose	0.11 L^{-1}
β_G	Clearance exponent (rate constant) for glucose	7.1e–4 s^{-1}
G_1	α_G/β_G	
D_β	EC_{50} of glucose at beta cells	7 mmol/L
α_I	Dilution factor ($1/V_D$) for insulin	0.2 L^{-1}
β_I	Clearance exponent for insulin	3.4e–3 s^{-1}
G_3	α_I/β_I	
D_R	EC_{50} of insulin at its receptor	1.6 nmol/L
G_E	Effector gain	50 s/mol
P	Constitutive endogenous glucose production	150 µmol/s

Source code

S functions for calculating structure parameters of the feedback loop

The following functions have been used to calculate SPINA-GBeta, SPINA-GR and SPINA-DI. They deliver vectorised results and are compatible with standard implementations of the statistical language S, e. g. with the R environment 3.5 or newer[2]:

```
SPINA.GBeta <- function(Insulin, Glucose)
# Insulin expected in pmol/l, Glucose in mmol/l
{
  pico.factor <- 1e12;
  mili.factor <- 1e3
  betaI <- 3.4e-3;
  alphaI <- 0.2;
  dBeta <- 7e-3;
  GBeta <- pico.factor * betaI * Insulin / pico.factor * (dBeta + Glucose /
    mili.factor) / (alphaI * Glucose / mili.factor);
  return(GBeta);
}

SPINA.GR <- function(Insulin, Glucose)
# Insulin in pmol/l, Glucose in mmol/l
{
  pico.factor <- 1e12;
  mili.factor <- 1e3;
  alphaG <- 0.11;
  betaG <- 7.1e-4;
  P0 <- 150e-6;
  DR <- 1.6e-9;
  GE <- 50;
  GR <- alphaG * P0 * (DR + Insulin / pico.factor) / (betaG * GE * Insulin /
    pico.factor * Glucose / mili.factor) - DR / (GE * Insulin / pico.factor)
    - 1 / GE;
  return(GR);
}

SPINA.DI <- function(Insulin, Glucose)
# Insulin in pmol/l, Glucose in mmol/l
{
  DI <- SPINA.GBeta(Insulin, Glucose) * SPINA.GR(Insulin, Glucose);
  return(DI);
}
```

Two-way sensitivity analysis

The following source code is implemented in SimulaBeta 3.1[3] to provide a disposition table based on two-way sensitivity analysis.

This unit is shown for illustration and explanation only. The code requires Free Pascal (version 3.0 or newer)[4], Lazarus (version 2.0 or newer)[5], and the CyberUnits Bricks Library (version 1.1 or newer)[6] to be compiled. Compiling demands additional units of SimulaBeta[3], which is available online along with source code from https://sourceforge.net/projects/simulabeta/ or https://doi.org/10.5281/zenodo.4922800.

```
unit SensitivityAnalysis;

{ SimulaBeta }

{ A simulator for insulin-glucose homeostasis }
{ Engine for sensitivity analysis }

{ Version 3.1.0 (Challenger) }

{ (c) Johannes W. Dietrich, 1994 - 2023 }
{ (c) Ludwig Maximilian University of Munich 1995 - 2002 }
{ (c) University of Ulm Hospitals 2002 - 2004 }
{ (c) Ruhr University of Bochum 2005 - 2023 }

{ Source code released under the BSD License }

{ See the file "license.txt", included in this distribution, }
{ for details about the copyright. }
{ Current versions and additional information are available from }
{ http://simulabeta.sf.net }

{ This program is distributed in the hope that it will be useful, }
{ but WITHOUT ANY WARRANTY; without even the implied warranty of }
{ MERCHANTABILITY or FITNESS FOR A PARTICULAR PURPOSE. }

{$mode ObjFPC}{$H+}

interface

uses
  Classes, SysUtils, SimulaBetaTypes, SimulationEngine;

type
  tTwoWaySensTable = array of array of TState;

function TwoWayTable(const xmin, xmax, ymin, ymax, resolutionx, resolutiony: real;
    const StrucPars: tParameterSpace; modX, modY: TParameter): tTwoWaySensTable;

implementation

function TwoWayTable(const xmin, xmax, ymin, ymax, resolutionx, resolutiony: real;
  const StrucPars: tParameterSpace; modX, modY: TParameter): tTwoWaySensTable;
var
  i, j, k: integer;
  maxi, maxj: integer;
  params: tParameterSpace;
  prediction: TPrediction;
begin
  params := StrucPars;
  prediction := PredictedEquilibrium(P0, 0, Z0, params);
  if prediction[0].G > 0 then
    k := 0
  else
    K := 1;
```

```
  maxi := trunc((xmax - xmin) / resolutionx) + 3;
  maxj := trunc((ymax - ymin) / resolutiony) + 3;
  SetLength(result, maxi, maxj);
  for i := 0 to maxi - 1 do
    for j := 0 to maxj - 1 do
      begin
        case modX of
          GR: params.GR := xmin + resolutionx * (i);
          GBeta: params.GBeta := (xmin + resolutionx * (i)) * PicoFactor;
        end;
        case modY of
          GR: params.GR := ymin + resolutiony * (j);
          GBeta: params.GBeta := (ymin + resolutiony * (j)) * PicoFactor;
        end;
        prediction := PredictedEquilibrium(P0, 0, Z0, params);
        result[i, j] := prediction[k];
      end;
end;

end.
```

The following additional type definitions and functions are defined in other units of SimulaBeta and are required for compiling this unit:

```
type

TState = record
  P, Q, R, G, S, I, M, N, W, Z: extended;
end;
TPrediction = array[0..1] of TState;

tParameterSpace = record
  alphaG, betaG, alphaI, betaI, GBeta, DBeta, GR, DR, GE: extended;
end;

tParameter = (alphaG, betaG, alphaI, betaI, GBeta, DBeta, GR, DR, GE);

TQRoots = array[0..1] of extended;


function SolveQuadratic(a, b, c: extended): TQRoots;
{ solves quadratic equation with parameters a, b and c }
begin
  Result[0] := -(b + sqrt(sqr(b) - 4 * a * c)) / (2 * a);
  Result[1] := -(b - sqrt(sqr(b) - 4 * a * c)) / (2 * a);
end;


function PredictedEquilibrium(P, W, Z: extended; StrucPars: tParameterSpace):
TPrediction;
var
    a, b, c, K1, K2: extended;
    G1, G3: extended;
begin
  G1 := StrucPars.alphaG / StrucPars.betaG; // Gain of ASIA element
  G3 := StrucPars.alphaI / StrucPars.betaI; // Gain of ASIA element
  Result[0].P := P;
  Result[1].P := P;
  Result[0].W := 0;
  Result[1].W := 0;
  Result[0].Z := Z;
  Result[1].Z := Z;

  { Solving for G: }
  with StrucPars do
  begin
    K1 := GE * GR * G3 * Z * GBeta / (DR + G3 * Z * GBeta);
    K2 := DR * DBeta / (DR + G3 * Z * GBeta);
```

```
    a := 1 + K1;
    b := K2 - G1 * Result[0].P;
    c := -G1 * K2 * Result[1].P;

    Result[0].G := SolveQuadratic(a, b, c)[0];
    Result[0].S := Z * GBeta * Result[0].G / (DBeta + Result[0].G);
    Result[0].I := G3 * Result[0].S;
    Result[0].M := GR * Result[0].I / (DR + Result[0].I);
    Result[0].N := GE * Result[0].M;
    Result[0].Q := Result[0].P / (1 + Result[0].N);
    Result[0].R := Result[0].Q;

    Result[1].G := SolveQuadratic(a, b, c)[1];
    Result[1].S := Z * GBeta * Result[1].G / (DBeta + Result[1].G);
    Result[1].I := G3 * Result[1].S;
    Result[1].M := GR * Result[1].I / (DR + Result[1].I);
    Result[1].N := GE * Result[1].M;
    Result[1].Q := Result[1].P / (1 + Result[1].N);
    Result[1].R := Result[1].Q;
  end;
end;
```

Supplementary Results

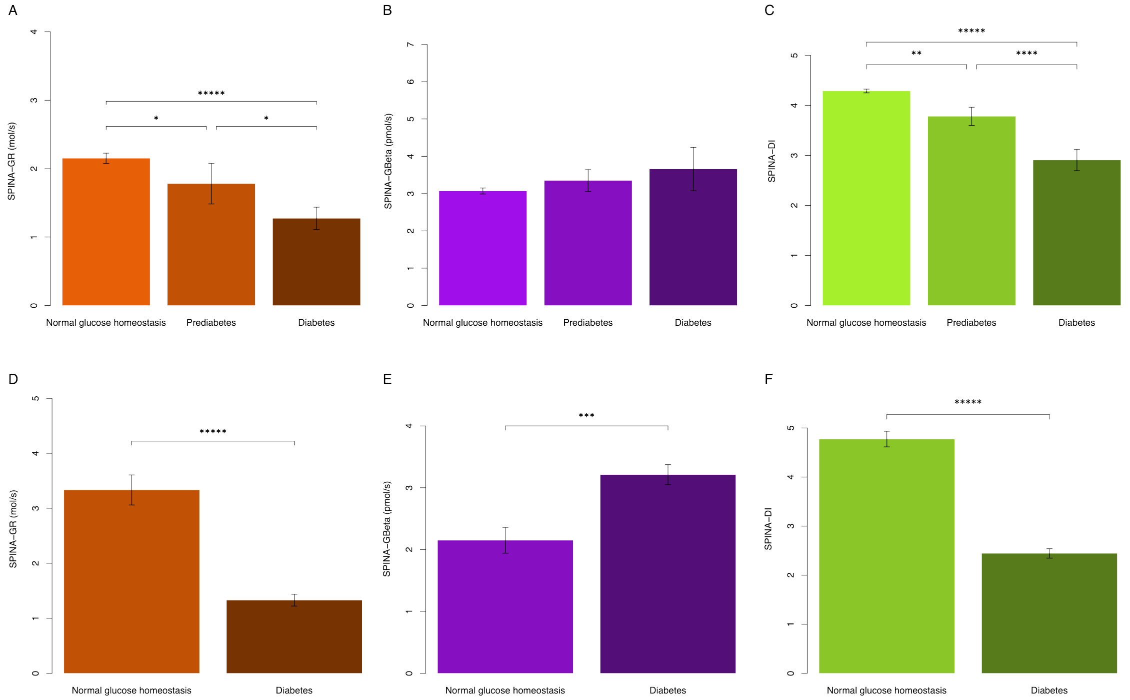

Fig. S1: Reconstructed insulin receptor gain (SPINA-GR), secretory capacity of pancreatic beta cells (SPINA-GBeta) and fasting-based disposition index (SPINA-DI) in the definition dataset (A to C) and the validation dataset (D to F).

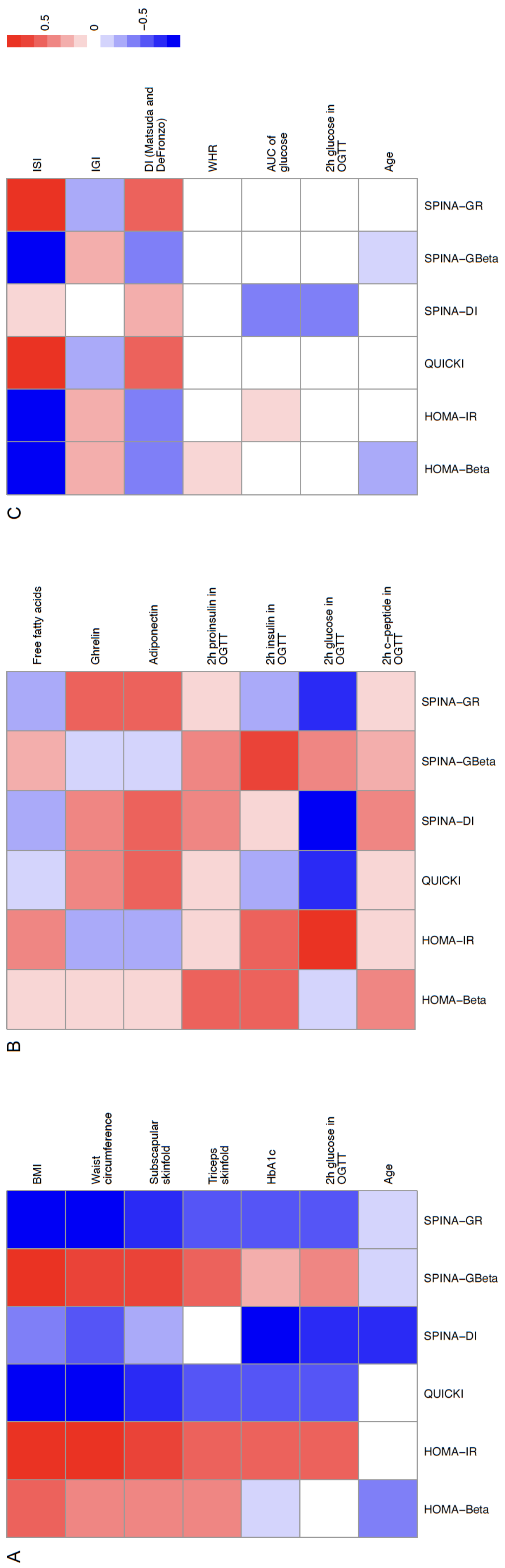

Fig. S2: Heatmaps of correlation networks in the definition (A), validation (B) and test (C) datasets. Shown in colour are significant correlations ($p < 0.05$) only, and the intensity of colour indicates the strength of negative (blue) or positive (red) correlation.

Table S2: Test-retest reliability and ergodicity of calculated biomarkers from repeated measurements. See Methods section for calculation of *e*. Larger Spearman's *rho* values denote higher reliability and higher *e* values represent lower ergodicity. ††† p < 1e–15

Parameter	*e*	Spearman's *rho*
HOMA-Beta	0.79	0.733†††
HOMA-IR	0.87	0.765†††
QUICKI	0.77	0.765†††
SPINA-GBeta	0.85	0.755†††
SPINA-GR	0.66	0.766†††
SPINA-DI	0.85	0.939†††

Supplementary References

1. Dietrich JW, Dasgupta R, Anoop S, et al. SPINA Carb: a simple mathematical model supporting fast in-vivo estimation of insulin sensitivity and beta cell function. *Sci Rep.* 2022;12(1):17659.
2. *R: A language and environment for statistical computing. R Foundation for Statistical Computing* [computer program]. Version 4.2.3. RRID:SCR_001905. Vienna, Austria: R Foundation; 2023.
3. *SimulaBeta* [computer program]. Version 3.1 . RRID: SCR_0219002021.
4. *Free Pascal: A 32, 64 and 16 bit professional Pascal compiler* [computer program]. Version 3.2.2. RRID: SCR_0143601993-2023.
5. *Lazarus: The professional Free Pascal RAD IDE.* [computer program]. Version 2.2.6. RRID: SCR_0143621993-2023.
6. *CyberUnits Bricks* [computer program]. Version 1.1.1. RRID:SCR_0143582020.
